# Recent Advances in the Development of Active and Intelligent Packaging Films Using Fruit Peel Powders

**DOI:** 10.3390/foods15010162

**Published:** 2026-01-03

**Authors:** Nianfeng Zhang, Bilal Ahmad, Fengfeng Xu, Jun Liu

**Affiliations:** College of Food Science and Engineering, Yangzhou University, Yangzhou 225127, China; nfzhang@yzu.edu.cn (N.Z.); mh24164@stu.yzu.edu.cn (B.A.); dx120240257@stu.yzu.edu.cn (F.X.)

**Keywords:** active packaging, film performance, fruit peel powders, intelligent packaging, preparation methods, structural characteristics

## Abstract

Fruit peels, a primary fruit processing by-product, are rich in biodegradable polymers (e.g., dietary fibers and proteins) and bioactive substances (e.g., polyphenols, essential oils, and pigments) that are suitable for producing active and intelligent packaging films. In recent years, there is a new trend to utilize fruit peels in the form of powders for film production, which aligns with circular economy principles. In general, fruit peel powders (FPPs) can function as rigid fillers and the polymer matrix in packaging films, forming FPP-filled and FPP-based films, respectively. These two film types exhibit distinct characteristics: FPP-filled films typically have a compact structure with strong molecular interactions, leading to superior mechanical and barrier properties. Conversely, FPP-based films often display a cracked structure with weaker molecular interactions, resulting in inferior mechanical and barrier properties. Despite these differences, both film types demonstrate excellent antioxidant and antimicrobial activities, pH sensitivity, and biodegradability, as well as considerable promise for active and intelligent packaging. This review comprehensively summarizes the preparation methods, structural characteristics, physical and functional properties, and active and intelligent packaging potential of both film types. It also features a multi-dimensional comparison of FPP-filled and FPP-based films’ performance and a discussion of their current challenges and future directions.

## 1. Introduction

With the evolution of the food industry, the demand for food packaging films is on the rise worldwide, which is accompanied by the generation of massive packaging waste. Nowadays, food packaging films are still largely made from fossil-based polymers, such as polyesters, polystyrene, polyvinyl chloride, polypropylene, and polyethylene terephthalate [1]. These polymers are non-biodegradable, and the resulting packaging can pose health risks if used inappropriately [2]. Driven by environmental and human health concerns, there is growing interest in the development of packaging films based on biodegradable and safe raw materials [3].

In recent years, fruit processing by-products (e.g., peels, pomace, and seeds) have emerged as promising biodegradable and renewable raw materials for packaging films [4,5]. These by-products are conventionally used as either animal feed or compost or even dumped in a landfill, and thus they are both underutilized and environmentally burdensome. As the primary by-product, fruit peels contain fully biodegradable biopolymers (e.g., soluble and insoluble dietary fibers, proteins, and lipids) ideal for constructing film matrices [6,7]. Additionally, they are an abundant source of bioactive substances (e.g., polyphenols, essential oils, and pigments) capable of enhancing film functionality, thereby allowing for their use in active and intelligent packaging [8,9]. Thus, developing packaging films from fruit peels significantly contributes to waste management and high-value utilization.

**Figure 1:**
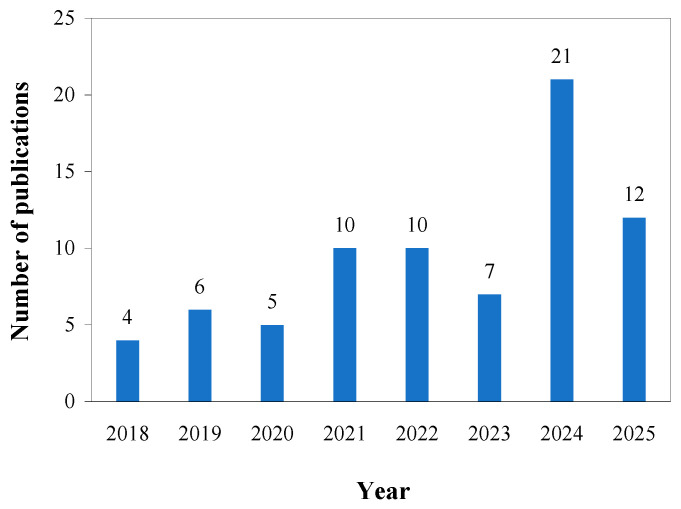
Statistics on the annual number of publications in the field of FPP-derived packaging films over the past decade. The data were obtained from the Scopus database using the keywords “film” and “fruit peel powder”.

Typically, biopolymers and bioactive substances are first extracted from fruit peels based on the biorefinery concept before being used to produce packaging films [10,11]. Although this method yields films from relatively pure compounds, it involves multiple extraction and purification steps that are time-consuming, costly, and difficult to scale up industrially. Meanwhile, the process still generates substantial residual waste, failing to meet the standard of a zero-waste process [12]. Recently, an alternative approach has emerged that fully utilizes fruit peels by drying and processing them directly into powders for film production. This novel method greatly simplifies the procedure, reduces costs, and aligns with the principles of circular economy and sustainable development. Specifically, compared to the packaging films made from the extracted biopolymers and bioactive substances, the films prepared directly from fruit peel powders (FPP) have simpler production procedure and lower cost. In contrast to conventional fossil-based packaging films, FPP-derived packaging films are inherently biodegradable, compostable and renewable. Furthermore, FPP-derived packaging films are non-toxic, ensuring safety for both food contact and environmental disposal. In recent years, researchers are increasingly focusing on the development of food packaging films using FPP, as evidenced by the growing number of papers in this field (Figure 1). However, to date, no specific review has summarized the recent advances in the development of packaging films using FPP.

In practice, FPP are typically blended with polymers in varying proportions to produce packaging films. When the proportion of FPP is lower than that of polymers, polymers form the continuous matrix, with FPP particles acting as fillers in the films. This type of films can be termed as FPP-filled films. Conversely, when the proportion of FPP exceeds that of polymers, FPP components (e.g., soluble and insoluble dietary fibers, proteins, and lipids) form the continuous matrix, with polymers acting as adhesive binders in the films. The type of films can be termed as FPP-based films. For the first time, this review comprehensively summarizes the preparation methods, structural characteristics, and physical and functional properties of both film types, alongside their applications in active and intelligent packaging. It also features a multi-dimensional comparison of FPP-filled and FPP-based films’ performance, along with a discussion of their current challenges and future directions.

## 2. Preparation and Characterization Methods of FPP

**Figure 2:**
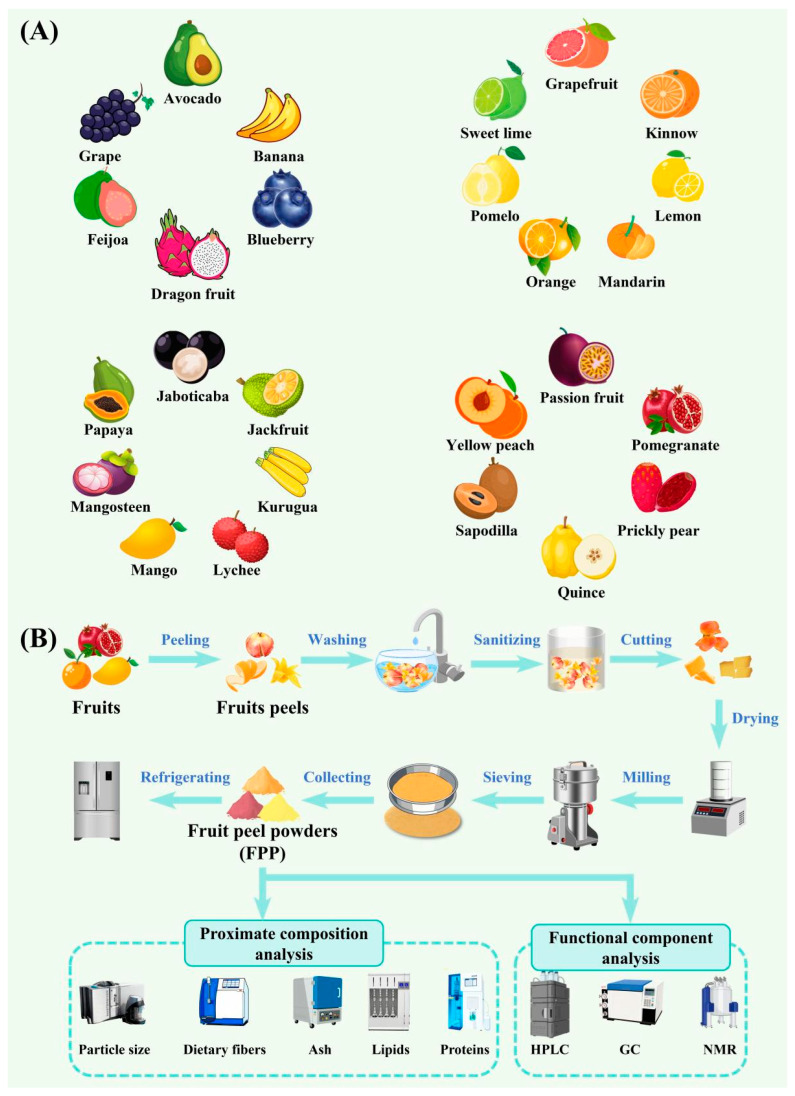
The sources of FPP used for the production of food packaging films (**A**) and the procedures for preparation and characterization of FPP (**B**).

As summarized in Figure 2A, numerous kinds of FPP have been used for the production of packaging films. The sources of FPP include avocado, banana, blueberry, dragon fruit, feijoa, grape, citrus fruits (e.g., grapefruit, kinnow, lemon, mandarin, orange, pomelo, sweet lime), jaboticaba, jackfruit, kurugua, lychee, mango, mangosteen, papaya, passion fruit, pomegranate, prickly pear, quince, sapodilla, and yellow peach. Among these sources, banana, dragon fruit, citrus fruits, mango and pomegranate are commonly employed in the production of FPP.

The preparation of FPP normally consists of several steps (Figure 2B). (1) The fruit peels from industrial processing by-products are typically washed with tap water, sanitized in a sodium hypochlorite solution, cut into small pieces, and dried to a moisture content of less than 10%. Notably, fruit peels contain some substances that are not desired for packaging films, such as soluble sugars and bitter compounds; desugaring and debittering treatments are sometimes applied during the pretreatment of fruit peels [13,14]. In addition, given that fruit peels contain several heat-sensitive substances, they should be treated by mild drying techniques, such as freeze drying and vacuum drying [15]. (2) The dried fruit peels are crushed using a grinder and sieved to obtain FPP with a uniform particle size. It should be noted that the particle size of FPP greatly affects the structural characterization and properties of packaging films [14,16]. However, in most studies, the particle size of FPP is not determined accurately using specialized instruments, such as a laser particle size analyzer, but is instead roughly estimated based on the mesh number of the sieves used. (3) The prepared FPP is stored at a low temperature for long-term preservation.

FPP has a very complex composition, comprising soluble dietary fibers (e.g., pectin and other soluble polysaccharides), insoluble dietary fibers (e.g., cellulose, hemicellulose, and lignin), proteins, lipids (e.g., essential oils), polyphenols (e.g., phenolic acids, flavonoids, stilbenes, and tannins), and pigments (e.g., anthocyanins, betacyanins and carotenoids) [9]. When used in packaging film production, the various components in FPP typically play different roles. For instance, the soluble and insoluble dietary fibers, proteins and lipids serve as the structural matrix of the films, while polyphenols, essential oils and pigments contribute additional functionalities. It is important to note that the composition of FPP is highly dependent on the fruit source, variety, geographical origin, growing conditions, and maturity. Therefore, the proximate composition (e.g., ash, soluble and insoluble dietary fibers, lipids, and proteins) and functional components (e.g., polyphenols, essential oils, anthocyanins, betacyanins, and carotenoids) in FPP should be thoroughly analyzed before preparing packaging films (Figure 2B). However, this key step is often overlooked in existing studies, and this omission makes it very difficult to analyze the constituent-function relationship of FPP-derived packaging films. Without proximate composition and functional component analyses, it is not feasible to explain why FPP-derived packaging films prepared in different studies have distinct performances. As summarized in Table S1, only few researchers have analyzed the proximate composition of FPP used in film production [16,17,18,19,20,21,22,23,24,25]. Furthermore, the total phenolic content of FPP is often quantified to assess the level of functional components. The detailed functional components in FPP are typically not analyzed in the original studies on film production; however, such information is available in relevant review articles [26,27,28,29,30,31].

## 3. Preparation Methods of FPP-Filled and FPP-Based Films

### 3.1. Film Components

**Figure 3:**
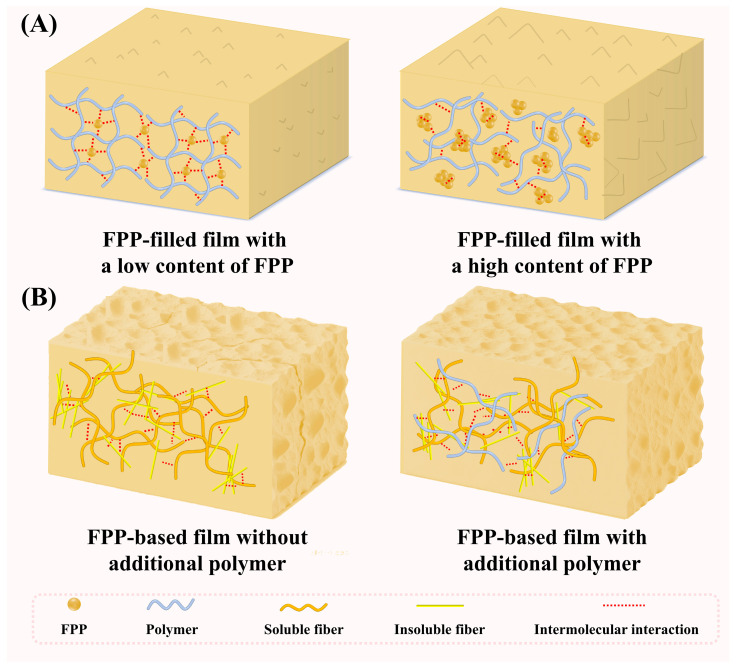
The diagrams showing the structural characteristics and molecular interactions of FPP-filled films (**A**) and FPP-based films (**B**).

As summarized in Table 1 and Table 2, FPP and polymers are two basic components of packaging films. Based on the function and proportion of FPP and polymers, packaging films can be classified as FPP-filled films and FPP-based films. In FPP-filled films, polymers form the continuous matrix, with FPP particles acting as fillers (Figure 3A). In FPP-filled films, FPP components (e.g., soluble and insoluble dietary fibers, proteins, and lipids) form the continuous matrix, with polymers acting as adhesive binders (Figure 3B). In FPP-filled films, the content of FPP is smaller than that of the polymers. Both synthetic and natural polymers can be used as the matrix of FPP-filled films. Compared with a single polymer, binary polymers can form stronger inter-molecular interactions, producing films with better physical properties [17,21,22,32,33,34,35,36,37]. Almost all kinds of FPP can be incorporated into polymer-based films as fillers. In FPP-based films, the content of FPP is equal to or greater than that of the polymers. It should be noted that fruits with soft peels, such as dragon fruit [38,39,40], pomegranate [41,42], mango [14,43,44], banana [23,44,45], peach [20], and citrus fruits [46,47,48,49,50,51,52,53], are normally rich in soluble dietary fibers with excellent film-forming capacity; therefore, they are highly suitable for preparing FPP-based films. In most cases, a single polymer is incorporated into FPP-based films to serve as a binder.

Except for FPP and polymers, different kinds of plasticizers, including glycerol, polyethylene glycol, sorbitol, and polyglycerine, are normally used to increase the ductility of FPP-filled and FPP-based films. Among these plasticizers, glycerol is the most widely used (Table 1 and Table 2). Moreover, cross-linking agents, including citric acid [14,36,37,50], CaCl_2_ [42,54], glutaraldehyde [89] and phosphoric acid [65], are sometimes added to elevate the mechanical properties of FPP-filled and FPP-based films. The incorporation of extra reinforcing agents, such as nanoparticles [18,46,53,58,73,82], clay platelets [62], and essential oils [43], can strengthen the functional properties of FPP-filled and FPP-based films.

Notably, the formulation of FPP-filled and FPP-based films greatly affects their structure and performance. So, a key direction for existing research is the formulation optimization of FPP-filled and FPP-based films, aiming for enhanced film performance. In most cases, the film formulation is tested using a single-factor experiment, where one factor (e.g., FPP content) is varied while the others (e.g., polymer and plasticizer contents) are kept constant. Single-factor experiment is helpful to assess the impact of an individual component on film performance. As summarized in Table 1, several studies have revealed that FPP content is a key factor affecting the physical and functional properties of FPP-filled films through single-factor experiments. In some cases, the total solid mass of the film components (i.e., FPP, polymer, and plasticizer) is kept constant, and the film formulation is optimized by varying the proportions of two or more components [17,19,32,35,36,39,54,83,94]. The film formulation can also be optimized using a central composite design, where the contents of two film components vary across three levels [18,21,46,59,60,85].

### 3.2. Preparation Methods

#### 3.2.1. Solvent Casting

**Figure 4:**
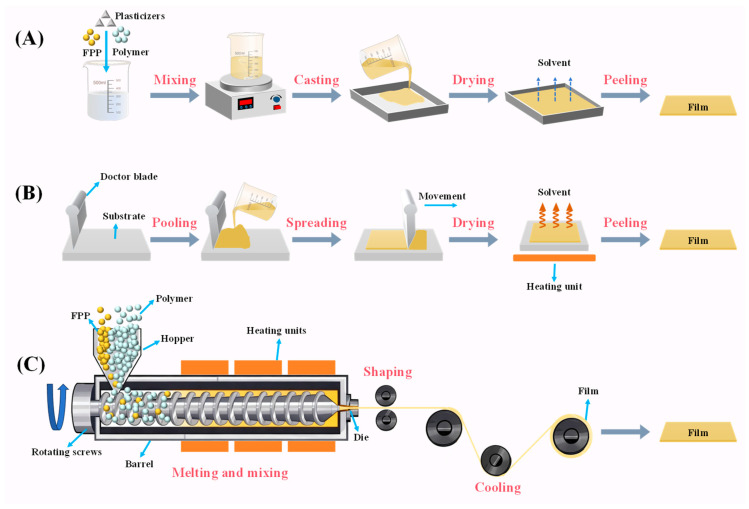
The preparation methods of FPP-filled and FPP-based films: solvent casting (**A**), knife coating (**B**), and extrusion (**C**).

Solvent casting is a simple and cost-effective method for the development of packaging films at a laboratory scale. As summarized in Table 1 and Table 2, solvent casting is the most widely used method for preparing FPP-filled and FPP-based films. The essential film components, including FPP, polymers, and plasticizers, are dissolved and combined in an appropriate solvent to obtain a film-forming solution. This solution is cast onto a smooth-surfaced mold and dried in a ventilated room. Once dried, the film is peeled off from the mold (Figure 4A). Solvent casting is a wet-processing technique that is typically operated in batch mode. Due to its low processing temperature, it is particularly useful for preparing films that contain heat-sensitive components, such as polyphenols [96]. In existing studies, film formulation, especially FPP content, has been demonstrated to be a key factor affecting the performance of the films (Table 1). Notably, FPP is composed of both soluble and insoluble components, which results in very limited compatibility with polymers. To solve this problem, several strategies have been developed, such as removing insoluble components from film-forming solution through centrifugation [13,81], reducing the particle size of FPP by intensive milling [14], as well as increasing the solubility of FPP by high-speed homogenization [16,49], ultrasound treatment [59], high-pressure homogenization [51], and macromolecular encapsulation [74].

#### 3.2.2. Knife Coating

Knife coating, also called blade coating, is a fast and simple method to fabricate FPP-filled and FPP-based films [23,38,56]. Similar to solvent casting, knife coating is a wet processing technique. As shown in Figure 4B, a knife coating apparatus comprises a doctor blade and a substrate. The process begins with a pool of film-forming solution being placed at one end of the substrate. The doctor blade, hanging over the solution, is then steadily traversed to the other end, uniformly spreading the solution for drying. To accelerate the drying process, a heating unit can be equipped beneath the substrate [97]. Knife coating offers the major advantage of producing uniform thin films over large surface areas, allowing for industrial-scale production of packaging films. The film thickness can be controlled by adjusting the doctor blade gap, its moving speed, and the rheological properties of the coating solution [97]. Notably, knife coating requires a film-forming solution with high viscosity [38]. In this regard, FPP with a high content of soluble dietary fibers is suitable for producing films through knife coating. For instance, dragon fruit and banana peel powders with high pectin contents have been used to fabricate FPP-based films via knife coating [23,38]. Future research should further investigate the influence of processing conditions on film performance.

#### 3.2.3. Extrusion

Extrusion is a continuous and highly efficient method to produce packaging films on an industrial scale. Unlike solvent casting and knife coating, extrusion is a dry processing technique that is based on the thermoplastic property of polymers [96]. Additionally, it requires lower energy consumption and a shorter processing time. However, extrusion has a higher cost in purchasing and maintaining specialized machinery. As shown in Figure 4C, polymers along with FPP particles (as fillers) and plasticizers are fed from a hopper into the barrel, where they undergo melting and mixing under the thermo-mechanical action of heating units and rotating screws. The molten film components are transported to the die, where they are shaped and cooled [35,36]. The extruded products are often processed into thin packaging films through blowing, injection, and compression molding. Polymers, such as poly(lactic acid) (PLA), polybutyrate adipate terephthalate (PBAT), polycaprolactone, and linear low-density polyethylene (LLDPE), have been blended with FPP and processed into FPP-filled films using extrusion followed by compression molding [35,61,69,91]. Film formulation and processing parameters (e.g., the feeding speed of raw materials, the configuration, rotating speed and length-to-diameter ratio of the screw, the heating profile of the barrel, and the size and shape of the die) critically influence film performance [35,36]. Notably, the raw materials used for extrusion are normally limited to heat-resistant substances. Given that the bioactive compounds (e.g., polyphenols) in FPP are sensitive to heat, the processing temperature and time of extrusion should be accurately controlled.

## 4. Structural Characteristics of FPP-Filled and FPP-Based Films

### 4.1. Microstructure

The microstructure of FPP-filled and FPP-based packaging films is frequently determined by ordinary optical microscope, scanning electron microscope and atomic force microscope. In addition, polarization microscope is occasionally applied to observe the distribution of crystalline constituents (e.g., cellulose) in the films [56,77]. As summarized in Table 1, the matrices of FPP-filled films are constituted by both natural and synthetic polymers. These polymers exhibit superior solubility and viscoelasticity in proper solvents, enabling formation of uniform and compact packaging films. FPP, when used as fillers, is capable of changing the microstructure of polymer-based films at a dose-dependent manner. As shown in Figure 3A, a low content of FPP can uniformly distribute in polymeric matrices and promote the compactness of the films. However, a high content of FPP tends to agglomerate in polymer-based films, thereby disrupting their uniformity and compactness [33,64,72,84]. In this context, the content of FPP in polymer-based films requires precise control. Meanwhile, developing effective methods to enhance FPP-polymer compatibility remains critical. One effective solution is to directly remove the insoluble components from film-forming solutions [13,81]. Another solution is to encapsulate FPP in macromolecular carriers (e.g., polysaccharides, proteins and lipids) [35,36,74].

Due to its high content of insoluble dietary fibers (e.g., cellulose, hemicellulose, and lignin), FPP exhibits an inferior film-forming property compared to pure polymers. In FPP-based films, the soluble dietary fibers (e.g., pectin) and protein of FPP form the matrix, while the insoluble components are randomly distributed within it (Figure 3B). During film formation, the soluble and insoluble components are likely to undergo phase separation, leading to structurally heterogeneous FPP-based films with internal cracks [23,38,46]. Therefore, FPP with fewer insoluble components is suitable for preparing FPP-based films with homogeneous microstructures [25,44,49]. Future studies need to screen proper raw materials for FPP-based films from diverse fruit resources. Except for the type of FPP, the particle size of FPP significantly impacts the microstructure of FPP-based films. Smaller FPP particles lead to films with smoother surfaces and more homogeneous microstructures compared to larger particles [14]. This indicated the microstructure of FPP-based films can be promoted by reducing the particle size of FPP. Additionally, the incorporation of polymers [39,46,48], plasticizers [38], cross-linking agents [50] and fillers [47,51] can improve the homogeneity and/or compactness of FPP-based films.

### 4.2. Molecular Interactions

The molecular interactions within FPP-filled and FPP-based packaging films are usually analyzed by Fourier Transform infrared (FT-IR) spectroscopy and X-ray diffraction (XRD). As summarized in Table 1, a variety of polymers have been employed as the matrix for FPP-filled films. These polymers comprise different functional groups, leading to distinct IR spectra in the resultant films. Furthermore, due to their capacity to form diverse inter- and intra-molecular interactions, films derived from different polymers often exhibit unique XRD patterns. Since FPP-filled films are mainly composed of polymers, their IR spectra and XRD patterns are very similar to those of pure polymer-based films. As shown in Figure 3A, when a low content of FPP is evenly distributed in polymer matrices, FPP can interact with polymer chains through hydrogen bonds and electrostatic interactions [33,64,84]. However, when excessive FPP is added to polymer matrices, FPP can automatically aggregate into clumps and the FPP-polymer interactions are weakened [55,64,76]. Therefore, the molecular interactions within FPP-filled films depend on the content of FPP in the films. Notably, FPP-filled films are characterized by the presence of unique FPP constituents. This is evidenced by distinct IR bands, such as C=O stretching band around 1730 cm^−1^ (from carbonyl groups in pectin and hemicellulose) and C=C stretching band around 1610 cm^−1^ (from aromatic rings in polyphenolic compounds) [33,66,67,76,86], and by diffraction peaks of crystalline substances like pectin and cellulose [55,64,76]. Both these characteristic spectral features typically intensify as the FPP content increases. Several studies have demonstrated that the interactions between FPP and polymers can be strengthened by adding reinforcing agents, such as nanoparticles [18,58,62,73,82] and cross-linking agents [36,37,54,65,89]. Nanoparticles typically act as multifunctional nano-fillers in the films. Due to their high specific surface area and abundant surface groups, nanoparticles can form strong physical interactions (e.g., hydrogen bonding, electrostatic attraction) with both FPP and polymer chains. Cross-linkers, on the other hand, can form covalent or ionic bonds with the reactive functional groups (e.g., hydroxyl and amino groups) of FPP and the polymer matrices, producing permanent and three-dimensional networks that significantly reduce polymer chain mobility. In addition, the encapsulation of FPP in macromolecular carriers (e.g., proteins, polysaccharides, and lipids) prior to film formation is another choice to strengthen the interactions between FPP and polymers [35,36,74]. The macromolecular carriers, selected for their inherent affinity with the bulk polymer matrices, can serve as compatibilizing agents and interact with polymer chains through hydrogen bonds and chain entanglement, which are thermodynamically more favorable than direct FPP-polymer interactions. 

Since the soluble components of FPP (e.g., pectin and proteins) constitute film matrices, the formation of FPP-based films mainly relies on the interactions between the soluble components [25,44]. However, the insoluble components in FPP often create a steric hindrance effect, which can negatively impact molecular interactions in FPP-based films (Figure 3B). Therefore, the molecular interactions within FPP-based films are normally weaker than those in FPP-filled films. In this context, different kinds of polymers have been added to FPP-based films to increase the proportion of soluble substances in the films [20,39,48]. The added polymers, acting as adhesive agents, can interact with FPP and elevate the compactness and mechanical strength of FPP-based films (Figure 3B). To date, numerous efforts have been undertaken to strengthen the molecular interactions in FPP-based films, such as pre-treating FPP through bleaching, high-speed homogenization and autoclaving [23], and incorporating plasticizers [38,44], cross-linking agents [14,42,50], and fillers [47,51,93] into the films. Pre-treatment of FPP primarily serves to modify the physicochemical state of FPP [23]. For instance, bleaching can expose more reactive hydroxyl groups and enhance hydrogen bonding potential. Homogenization can reduce the particle size and increase the specific surface area of FPP for better interfacial contact. Autoclaving can disrupt cellular structures and promote the release or generation of more compatible polymeric fragments [23]. Incorporating plasticizers works indirectly by increasing the free volume and chain mobility of polymer matrices, which facilitate a more uniform dispersion of FPP in the films [38,44]. Incorporating cross-linking agents is helpful to create permanent covalent or ionic bridges between the functional groups of FPP [14,42,50]. In addition, incorporating fillers is capable of reducing the void space within in the films [40,47,51,93].

## 5. Physical Properties of FPP-Filled and FPP-Based Films

### 5.1. Hydrophobicity

Hydrophobicity is essential for packaging films to maintain their stability in highly humid environments. The hydrophobicity of FPP-filled and FPP-based films is normally evaluated by measuring their moisture content (MC), water solubility (WS), swelling ratio (SR) and water contact angle (WCA). For FPP-filled films, their hydrophobicity mainly depends on the nature of the polymers. As summarized in Table 1, hydrophilic polymers including polyvinyl alcohol (PVA), gelatin, chitosan and starch are frequently selected to prepare FPP-filled films through casting, whereas hydrophobic polymers including PLA, polycaprolactone and LLDPE are preferred to prepare FPP-filled films through extrusion. Therefore, selecting proper polymers and film preparation method are important for producing FPP-filled films with ideal hydrophobicity. FPP, composed of both hydrophobic and hydrophilic components, can influence the hydrophobicity of polymer-based films in different aspects: (1) The MC, WS and SR of polymer-based films are typically reduced by FPP, as FPP-polymer interactions decrease the polymer’s affinity for water [13,64,67]. Meanwhile, the incorporation of FPP increases the proportion of insoluble components in polymer-based films [64,66,81]. (2) The WCA of polymer-based films is normally increased by FPP, as FPP significantly elevates the surface roughness of the films [63,71,81]. (3) In addition, the hydrophobicity of polymer-based films often increases with increasing FPP content [13,64,66,81]. Notably, for FPP-filled films with hydrophilic polymers as the matrices, improving their hydrophobicity remains a significant challenge. Studies have demonstrated that the incorporation of montmorillonite clay platelets [62], cross-linking agents [37,54,89] and nanoparticles [58] can enhance the hydrophobicity of FPP-filled films. Another option is to laminate FPP-filled films with commercially available fossil-based films, which can provide hydrophobic barriers [79].

As compared to FPP-filled films, FPP-based films contain a higher proportion of insoluble components and are thereby more hydrophobic. Since FPP from different sources has varying constituents, the hydrophobicity of FPP-based films depends on the type of FPP [25,44]. Theoretically, FPP with more insoluble components tends to produce films with higher hydrophobicity. Paradoxically, FPP containing a high content of insoluble components is detrimental to film formation. Therefore, when screening suitable raw materials for FPP-based films, it is necessary to balance the hydrophobicity and structural integrity of the films. In practice, FPP is often blended with hydrophilic polymers to prepare FPP-based films (Table 2). Although these polymers are hydrophilic, they effectively enhance the hydrophobicity of FPP-based films by forming compact internal structures [20,48]. In contrast, the incorporation of plasticizers often reduces the hydrophobicity of FPP-based films, and the extent of this reduction is influenced by the type and content of the plasticizers used [14,42,43,44,48]. When hydrophobic substances like essential oils [43], *N*-(2-amino-ethyl)-3-aminopropyltrimethoxysilane [14], sugarcane bagasse fiber [93], wheat straw, and rice husk [47] are incorporated into film-forming solutions, the resulting FPP-based films normally exhibit improved hydrophobicity. Some recent studies have demonstrated that the hydrophobicity of FPP-based films can be enhanced by direct immersion in waxes, which forms a hydrophobic barrier around the films [24,45].

### 5.2. Mechanical Properties

The mechanical properties of packaging films play a critical role in ensuring effective food protection during food packaging and storage. Tensile strength (TS), elongation at break (EAB) and Young’s modulus (YM) are commonly measured to judge the mechanical properties of FPP-filled and FPP-based films. In addition, other mechanical property-related indices, such as penetration force [85], folding endurance [17], and heat sealing ability [86], are occasionally tested for the films. The mechanical properties of FPP-filled and FPP-based films are closely associated with the microstructure of the films and the interactions of film components. As shown in Figure 3A, FPP-filled films normally exhibit compact structures with good mechanical properties, as polymers have superior film-forming ability and strong inter- and intra-molecular interactions. FPP can interact with polymer chains and change their interactions and spatial arrangement. As summarized in Table 1, the mechanical properties of FPP-filled films are affected by the content of FPP in the films. A low content of FPP has good compatibility and interfacial adhesiveness with polymer matrix. The rigid FPP particles are evenly dispersed in polymer matrix and establish inter-molecular interactions with polymer chains, producing stiffer films with elevated TS and YM. In contrast, the interaction and mobility of polymer chains are weakened by FPP particles, and thus the films become more brittle and exhibit declined EAB. When excessive FPP particles are added, they can gather together and the formed FPP agglomerates disrupt the compactness of the films and reduce the stress transfer efficiency from filler to polymer matrix. As a result, the TS and YM of the films tend to decline [55,56,64,66,67,72,77,80]. Some studies have revealed that the mechanical properties of FPP-filled films are influenced by the type of FPP [68] and the content of plasticizers [37]. Notably, the mechanical properties of FPP-filled films can be elevated by incorporating other fillers, such as montmorillonite clay platelets [62], nanoparticles [58,73,82] and cross-linking agents [37,54,89]. Additionally, the encapsulation of FPP in macromolecular carriers can enhance the comparability between FPP and polymer matrix, thereby elevating the mechanical properties of FPP-filled films [74].

As compared with FPP-filled films, FPP-based films typically exhibit lower mechanical properties. Especially when FPP is used as the only film component, the resulting films have inadequate strength and flexibility [20,38,46,47]. On one hand, the soluble and insoluble components in FPP tend to phase separate, producing films with heterogeneous and loose structures. On the other hand, the molecular interactions within FPP-based films are weaker than those in FPP-filled films (Figure 3B). Some studies have revealed that the type of FPP greatly impacts the mechanical property of the films, where FPP with more insoluble components produces films with lower mechanical properties [25,49]. Interestingly, the particle size of FPP is negatively related to the mechanical properties of FPP-based films, as smaller FPP have bigger specific surface area to interact with other film components [14]. In addition, bleaching and autoclaving of FPP can modify its insoluble components and improve the mechanical properties of the resulting films [23]. In practice, polymers are normally incorporated into FPP-based films to enhance mechanical properties, as they function as adhesives for the FPP components. Similarly, plasticizers are added to these films to promote flexibility (Table 2). Therefore, the type and content of polymers and plasticizers significantly affect the mechanical property of FPP-based films [14,38,43,44,48]. The mechanical property of FPP-based films can be effectively elevated by incorporating cross-linking agents, such as CaCl_2_ and citric acid [14,42,50]. Oppositely, when FPP-based films are coated with hydrophobic waxes, they exhibit reduced mechanical property [24,45]. This is because the coated waxes are loosely attached to the films. A recent study has provided a solution by heat-pressing the wax-coated films to increase wax-film adhesion [98].

### 5.3. Barrier Properties

External environmental factors (e.g., light, water vapor and oxygen) can detrimentally affect food quality, and thus packaging films should possess barrier properties against these factors. The barrier properties against light, moisture and oxygen are typically evaluated by measuring light transmittance (LT), water vapor permeability (WVP) and oxygen permeability (OP), respectively. Notably, a fundamental methodological flaw in packaging film research is the lack of standardized testing protocols for key barrier properties like WVP and OP. Since the measurements of WVP and OP normally involve several unstandardized test parameters, such as test temperature, relative humidity, and test duration, the reported WVP and OP values in different studies are frequently incomparable. Therefore, it is imperative that future studies adopt the established international standards (e.g., ASTM or ISO methods) for these measurements.

For FPP-filled films, their barrier properties are closely associated with film structural integrity and FPP particle distribution. Because of their compact inner structures, pure polymer-based films typically exhibit certain barrier properties against light, water vapor and oxygen. FPP can influence the barrier ability of polymer-based films through various mechanisms. (1) Polyphenols and lignin in FPP possess aromatic ring structures capable of strongly absorbing UV-Vis radiation. Meanwhile, FPP particles dispersed in the films can scatter light propagation. Therefore, the LT of polymer-based films is always reduced by FPP [33,63,66,77,84]. (2) FPP particles can enter the free volume of the polymer-based films and impede the diffusion of water vapor and oxygen gas. Consequently, the WVP and OP of polymer-based films are normally reduced by FPP incorporation [18,58,68,86]. (3) The barrier properties of polymer-based films typically increase with increasing FPP content. However, excessive FPP may cause particle aggregation, thereby compromising film compactness. As a result, the WVP and OP of the films increase beyond an optimal FPP concentration [13,22,64,76,82]. Apart from the FPP content, both the type and particle size of FPP significantly affect the barrier properties of the films [16,68]. For instance, a smaller FPP particle size creates more complex moisture pathways, which leads to a lower WVP [16]. Studies have demonstrated that the barrier properties of FPP-filled films can be synergistically improved by incorporating nanofillers, such as montmorillonite clay platelets [62] and nanoparticles [18,58].

As compared with FPP-filled films, FPP-based films typically exhibit higher light barrier property but lower water vapor and oxygen barrier properties. The superior light barrier property of FPP-based films is attributed to abundant polyphenols and lignin in FPP, whereas their poorer barrier properties against water vapor and oxygen are due to loose film structures. Thus, few studies have reported on the WVP and OP of FPP-based films. Existing studies have revealed that the type of FPP significantly influences the barrier properties of FPP-based films [25,49]. FPP with fewer insoluble components tends to produce films with more compact structures and better water vapor and oxygen barrier properties [49]. Additionally, bleaching and autoclaving treatments on FPP can modify its insoluble components, enhancing both the structural integrity and water vapor barrier properties of the resulting films [23]. In most cases, different kinds of polymers are incorporated into FPP-based films to create compact structures, which enhance the water vapor and oxygen barrier properties of the films [46,48]. Furthermore, the co-incorporation of fillers, such as sugarcane bagasse fiber [93], wheat straw powder, and rice husk powder [47], can synergistically improve the water vapor barrier property of FPP-based films. Interestingly, wax coatings remarkably improve the water vapor barrier properties of FPP-based films by forming a hydrophobic surface [24,45].

### 5.4. Thermal Properties

The thermal properties (e.g., thermal decomposition and thermal stability) of FPP-filled and FPP-based films are typically analyzed by thermogravimetry and differential scanning calorimetry (DSC), with thermogravimetry being more frequently applied. The thermal decomposition of pure polymer-based films involves several stages, including the evaporation of free water molecules, the decomposition of low-molecular-weight substances (e.g., plasticizers and bioactive compounds), the breakdown of polymer chains, and the carbonization of the residue [76]. As indicated in Table 1, the thermal properties of FPP-filled films are affected by the particle size of the FPP. When small-sized FPP particles are incorporated into polymer-based films, they normally elevate the thermal stability of the films. This improvement is due to the presence of rigid FPP particles and the synergistic interactions between FPP and polymers [18,22,56,58,86]. However, when large-sized FPP particles are added to polymer-based films, they have an adverse impact on the thermal stability of the films [61,63]. Some studies have demonstrated that the thermal stability of FPP-filled films increases with increasing FPP content [13,34,76,83].

To date, few studies have reported the thermal properties of FPP-based films. Existing studies have demonstrated that the thermal stability of FPP-based films is influenced by the types of FPP and plasticizers [14,25,44,49]. However, the particle size of FPP has little impact on the thermal stability of FPP-based films [14]. The incorporation of polymers [20,46] and hydrophobic agents [50] can improve the structural compactness and thermal stability of FPP-based films. In addition, wax coatings can provide a protective barrier and elevate the thermal stability of the films [24].

## 6. Functional Properties of FPP-Filled and FPP-Based Films

### 6.1. Antioxidant and Antimicrobial Activities

**Figure 5:**
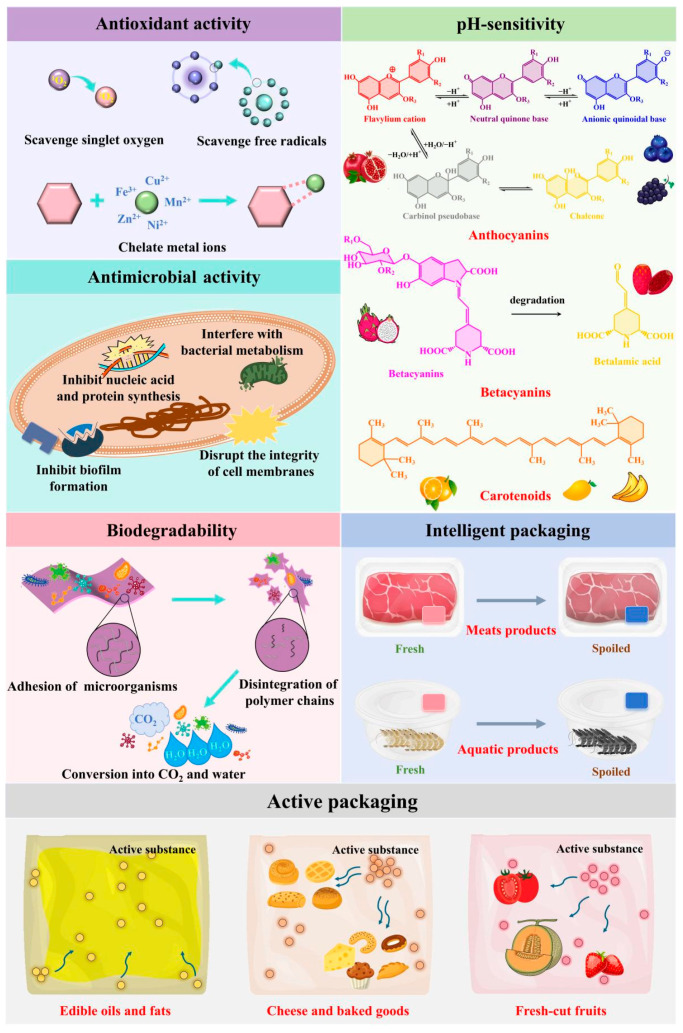
The antioxidant, antimicrobial, pH-sensitive, biodegradable mechanisms and application forms of the films in active and intelligent packaging.

Food products are highly perishable due to oxidation and microbial contamination. In recent years, various antioxidant and antimicrobial packaging films have been developed by incorporating preservatives. Considering that synthetic preservatives are unfriendly to human health and the environment, natural source-derived preservatives, generally recognized as safe (GRAS), are preferred additives for food packaging films. Fruit peels are natural sources rich in bioactive compounds (e.g., polyphenols, ascorbic acid, essential oils, and pigments) with antioxidant and antimicrobial activities [9]. As summarized in Table 1 and Table 2, both FPP-filled and FPP-based films exhibit good antioxidant and antimicrobial activities. It is generally accepted that polyphenols in FPP are primarily responsible for the antioxidant and antimicrobial activities of FPP-filled films [13,22,72,79,81,84]. As shown in Figure 5, the main antioxidant mechanisms of polyphenols involve their ability to scavenge reactive oxygen species (e.g., singlet oxygen, O_2_^•−^, H_2_O_2_, and •OH) and chelate metal ions [99]. Meanwhile, polyphenols exert antimicrobial activity through multiple pathways, including disrupting the integrity of cell membranes, inhibiting biofilm formation, interfering with bacterial metabolism, and inhibiting nucleic acid and protein synthesis [100]. The antioxidant and antimicrobial activities of polyphenols depend on their structural characteristics, particularly the number and position of phenolic hydroxyl and other substituent groups [101]. Notably, apart from polyphenols, other bioactive compounds in FPP (e.g., essential oils and pigments) also possess antioxidant and antimicrobial activities, which are often neglected in existing studies. These bioactive compounds often exhibit synergistic antioxidant and antimicrobial effects in FPP, which is a key advantage of using the whole powder over the isolated extracts.

In FPP-filled films, only few polymer matrices (e.g., chitosan) possess inherent antioxidant and antimicrobial activities. Therefore, FPP is the main antioxidant and antimicrobial agents in FPP-filled films. Numerous studies have demonstrated that the antioxidant and antimicrobial activities of FPP-filled films increase with increasing FPP content [13,22,64,66,72,77,79,80,81,84]. Additionally, different types of FPP have distinct polyphenolic contents and compositions, which significantly affect the antioxidant and antimicrobial activities of FPP-filled films [44,68,79]. The antimicrobial activity of FPP-filled films can be further strengthened by incorporating other antimicrobial agents, such as montmorillonite clay platelets [62] and nanoparticles [58,82]. However, the toxicological effects of these nano-sized antimicrobial agents should be carefully considered before use.

FPP-based films exhibit higher antioxidant and antimicrobial activities than FPP-filled films, not only because they contain a higher proportion of FPP but also because their loose structures allow polyphenols to be released more easily. Studies have revealed that the type of FPP has a big impact on the antioxidant and antimicrobial activities of FPP-based films [25,44,49]. Small-sized FPP-filled films exhibit higher antioxidant and antimicrobial activities than large-sized ones, as polyphenols are more readily released from the smaller FPP particles [14]. Interestingly, blanching treatment is capable of protecting polyphenols in FPP by inactivating polyphenol oxidase and peroxidase, thereby producing films with improved antioxidant and antimicrobial activities [23]. The antioxidant and antimicrobial activities of FPP-based films can be further elevated by incorporating essential oils [43], nanoparticles [46,53], tea polyphenol [51], wheat straw powder, and rice husk powder [47]. It is worth noting that FPP-based films are porous, allowing them to absorb moisture and oxygen which makes polyphenols in FPP very unstable. Therefore, FPP-based films are normally less stable than FPP-filled films. In this context, it is necessary to provide protection for FPP-based films. Some recent studies have revealed that wax coatings can obstruct moisture permeation and increase the stability of polyphenols in the films [24,45]. To overcome the steric hindrance caused by insoluble components in FPP-based films, several novel techniques can be employed. For instance, pretreating FPP with enzymes such as cellulase and pectinase can degrade the cell wall structure, converting insoluble fibers into water-soluble molecules [102]. Alternatively, a promising composite design involves selectively extracting key components (e.g., pectin, and cellulose) from FPP and then processing them into packaging films [103].

### 6.2. pH Sensitivity

Food spoilage is typically accompanied by the release of acidic or alkaline gases, such as CO_2_, H_2_S, and biogenic amines. Natural pigments, such as anthocyanins, betacyanins, and carotenoids, are pH-sensitive substances that are widely distributed in fruit peels. When these pigments encounter acidic or alkaline gases, the resulting pH change can alter their molecular structures and colors [104]. As shown in Figure 5, anthocyanins are water-soluble and polyphenol-type pigments that are broadly distributed in fruits with red, blue, and purple peels, such as blueberries, grapes, and pomegranates. In general, anthocyanins turn red/pink under acidic conditions and blue/green/yellow under alkaline conditions. This color change is associated with the transformation among different forms of anthocyanins, such as the flavylium cation, the neutral quinone base, the anionic quinoidal base, the carbinol pseudobase, and the chalcone [101]. Betacyanins are water-soluble pigments that are found in fruits with red-violet peels, such as dragon fruits and prickly pears. Betacyanins exhibit a stable red-violet color under acidic conditions but turn yellow under alkaline conditions. The alkali-induced color changes are caused by the degradation of betacyanins into betalamic acid [105]. Carotenoids are also pH-sensitive pigments that are typically found in fruits with yellow or orange peels, such as citrus, bananas, peaches, apricots, and mangoes. Unlike anthocyanins and betacyanins, carotenoids are fat-soluble. They become clearer under extreme acidic conditions (pH < 3) and turn more turbid under extreme alkaline conditions (pH > 12), which is attributed to the protonation and cis-trans isomerization of carotenoids [106]. Therefore, the pH sensitivity of carotenoids is weaker than that of anthocyanins and betacyanins.

As summarized in Table 1 and Table 2, anthocyanin- and betacyanin-rich FPP has been used to develop packaging films with the ability to indicate food freshness through color changes [24,33,38,39,57,60,63,78]. In contrast, carotenoid-rich FPP has not been used to develop intelligent packaging films. Most existing studies have reported that the pH sensitivity of FPP-filled films increases with increasing FPP content, as the color changes in the pigments become more obvious at higher concentrations [33,38,63,78]. In this respect, FPP-based films exhibit better pH sensitivity than FPP-filled films. It is worth noting that, on the one hand, the pigment content and composition vary greatly in different fruit peels, yet the pH sensitivity of films derived from these different sources has rarely been compared [57]. On the other hand, natural pigments are susceptible to factors like oxidation, high humidity, light exposure, and high temperature, which typically cause color fading [101]. FPP-based films, due to their loose inner structures, can easily absorb oxygen and moisture. Therefore, the color stability of FPP-based films is worse than that of FPP-filled films. Future studies are needed to improve the stability of natural pigments in both types of films through techniques such as encapsulation and copigmentation.

### 6.3. Biodegradability

Biodegradability, the breakdown of food packaging films by microorganisms, is a desirable property. The biodegradation of the films typically comprises three steps: the adhesion of microorganisms to the films, the disintegration of polymer chains into small molecules, and the conversion of these molecules into CO_2_ and water (Figure 5). For FPP-filled films, their biodegradability is highly related to the hydrophilicity of polymer matrices. Generally, hydrophilic polymer matrices hold high water absorption and microbial adhesion, leading to good biodegradability. In contrast, most traditional fossil-based hydrophobic polymer matrices (e.g., LLDPE) are resistant to water absorption and microbial adhesion, and thus are highly resistant to biodegradation [69]. Instead, bio-based hydrophobic polymer matrices (e.g., PLA and thermoplastic starch) are more recommended to prepare FPP-filled films due to their biodegradability and industrial applicability [32,61]. Studies have shown that the biodegradability of polymer-based films is typically accelerated after incorporating FPP [55,61,67,71,83,86,87]. This is because FPP particles increase the roughness of the films and facilitate microbial adhesion. The films filled with a high content of FPP often exhibit better biodegradability, possibly due to the formation of loose inner structures [67,71,83,87].

FPP-based films are expected to have better biodegradability than FPP-filled films, not only because the films are mainly composed of bio-based raw materials but also because the films possess looser inner structures that facilitate water adsorption and microbial adhesion. To date, only a few studies have reported the biodegradability of FPP-based films [24,44,47,49]. The biodegradability of FPP-based films is affected by the type of FPP [44,49]. The incorporation of fillers, such as wheat straw and rice husk powders, retards the degradation of FPP-based films, probably due to the formation of compact inner structures [47]. Meanwhile, wax coating can inhibit water adsorption and microbial adhesion, thereby slowing down the biodegradation rate of the films [24].

## 7. Applications of FPP-Filled and FPP-Based Films in Food Packaging

### 7.1. Active Packaging

Nowadays, packaging films integrated with antioxidant and antimicrobial agents are emerging as one of the main active packaging techniques. Since FPP is rich in bioactive substances (e.g., polyphenols, essential oils, and pigments) with antioxidant and antimicrobial activities, FPP-filled and FPP-based films are considered promising active packaging materials. As summarized in Table 1 and Table 2, FPP-filled and FPP-based films have been used for the active packaging of diverse food categories. These include dairy products (e.g., cheese), baked goods (e.g., bread, muffin), oils and fats (e.g., edible oils, lard), fruits (e.g., plum, grape, fresh-cut apple, fresh-strawberry), fruit juices (e.g., apple juice), and vegetables (e.g., bean sprout). As displayed in Figure 5, the films are typically made into sealed pockets for packaging fruit juices, oils and fats [20,25,49,51,64,66,74]. They are effective in retarding oxidation by releasing antioxidant agents while blocking light and oxygen. When used for packaging cheese, baked goods, fruits and vegetables, the films typically serve as wrapping or covering materials, extending the shelf life by inhibiting oxidation and preventing microbial contamination [16,19,42,64,75,86,90]. Additionally, edible coating is another approach to preserve fruits. This is achieved by dipping the fruits into film-forming solutions, which then dry into intact protective barriers [43,65]. In the future, FPP-filled and FPP-based films could also be used for the active packaging of meat products. FPP-based films, due to their porous internal structures, are particularly suitable as absorbent pads for meat packaging. Notably, the application of FPP-filled and FPP-based films in active packaging mainly relies on their antioxidant and antimicrobial activities. In practice, these films are designed to maintain close contact with food products to ensure the controlled and durable release of bioactive substances. However, only a limited number of studies have systematically investigated the release kinetics of bioactive substances in realistic food models [24,35,84]. This insufficient investigation leaves several pivotal questions unanswered: the release rates and mechanisms under varied food conditions (e.g., pH, fat/water content), the duration of effective concentration at the food surface, and the potential for premature depletion or excessive initial migration. Without a thorough kinetic understanding, the claimed “active” function remains largely speculative and unoptimized. Therefore, future research should quantify the release kinetics of bioactive substances across a representative spectrum of food simulants and real matrices. This will enable the rational design of packaging films with tailored release profiles to match specific food preservation needs.

### 7.2. Intelligent Packaging

Intelligent packaging, an emerging food packaging technique, is designed to provide the consumer with real-time information about the freshness or spoilage state of food products [101]. Packaging films containing pH-sensitive pigments are a form of intelligent packaging material that can indicate the freshness of food products via different colors [104]. As shown in Figure 5, the films are typically integrated into conventional packaging as interior labels to monitor changes in the acidic and alkaline metabolites of food products. The films are normally attached to the internal surface of packaging containers, without contacting food products. In some cases, the films are wrapped around the food products with direct contact [60]. In this case, pigments might migrate from the films into food products, resulting in undesirable coloration. As summarized in Table 1 and Table 2, FPP-filled and FPP-based films containing anthocyanins or betacyanins have been used to indicate the freshness of meat products (e.g., pork, chicken, lamb) and aquatic products (e.g., shrimp). This functionality is based on the color changes in anthocyanins and betacyanins in alkaline conditions created by biogenic amines. Studies have demonstrated that the color changes in the films correlate well with parameters indicative of quality deterioration in food products, such as pH, total viable count (TVC) and total volatile base nitrogen (TVB-N) [24,33,38,39,40,60,63,78]. It is worth noting that FPP-filled and FPP-based films containing anthocyanins can change color under acidic conditions, making them promising CO_2_ sensors for indicating the freshness of fruits and vegetables in the future.

## 8. Current Challenges of FPP-Filled and FPP-Based Films

Raw material variability: The standardized production and quality control of films are challenged by raw material complexity. The performance of FPP-filled and FPP-based films largely depends on the material characteristics of FPP, in particular its proximate composition and functional components. Notably, the material characteristics of FPP are influenced by the fruit source, variety, geographical origin, growing conditions, and maturity. To date, only a few studies have compared the performance of packaging films prepared from FPP of different sources [44,57,68,79,89,91]. In addition, the effects of other factors (e.g., the variety and geographical origin of FPP) on film performance are frequently neglected [25,49].

Production unscalability: In existing studies, FPP-filled and FPP-based films are mainly produced by the laboratory-scale solvent casting method (Table 1 and Table 2), which is operated in a discontinuous way and is inefficient for large-scale production.

Inherent performance flaws: When comparing film performance, FPP-filled and FPP-based films are found to have their own strengths and weaknesses (Table 3). FPP-filled films, due to their uniform and compact inner structures as well as strong molecular interactions, exhibit good mechanical, barrier and functional stability. Notably, the hydrophobicity and biodegradability of FPP-filled films are influenced by the polymer matrix source. Using hydrophilic polymers typically results in films with low hydrophobicity and poor FPP-polymer compatibility, while using fossil-based polymers generally leads to low biodegradability. Moreover, the application of FPP-filled films is limited by unsatisfactory functionality (e.g., limited antioxidant/antimicrobial activities and pH sensitivity) and high polymer cost, primarily due to their low FPP content. In contrast, FPP-based films, owing to their high FPP content, exhibit excellent light barrier performance, strong antioxidant/antimicrobial activities, pH sensitivity, and good biodegradability. However, they suffer from structural discontinuity, poor mechanical strength, low barrier properties against water vapor and oxygen, and limited functional stability.

Regulatory hurdles: The commercial-scale application of FPP-filled and FPP-based films faces significant regulatory hurdles, primarily due to the absence of clear frameworks for certification and standardization.

## 9. Future Perspectives of FPP-Filled and FPP-Based Films

Material science focus: Considerable effort must be devoted to selecting suitable FPP as the raw material for film production. Meanwhile, it is essential to investigate the relationship between FPP material characteristics and film performance through correlation analysis, thereby enabling the rational design of high-performance films.

Process engineering focus: Knife coating and extrusion are industrial-scale film production methods that deserve wide application in the future. Moreover, the formulation of film components and the processing parameters of knife coating and extrusion should be optimized. Special attention should be paid to the rheological properties of film-forming solutions used for knife coating and the thermal stability of bioactive substances in FPP used for extrusion.

Performance promotion focus: Future efforts are required to enhance the FPP-polymer compatibility and impart hydrophobicity to hydrophilic polymer-based films through different means, such as modifying FPP through chemical, physical and enzymatic methods, removing insoluble components from the film-forming solution, reducing the particle size of FPP, encapsulating FPP in macromolecular carriers, and incorporating proper nanofillers, cross-linking agents, and hydrophobic agents.

Application-focused research: Systematic studies on release kinetics, migration, and real-food efficacy trials are urgent. Moreover, future work must include sustainability & life cycle assessment (LCA) on the developed films to validate their environmental benefits compared to conventional or even other bio-based films. Meanwhile, it is necessary to establish production standards and strengthen regulatory support for these films.

## 10. Conclusions

FPP, when blended in different proportions with polymers, can be used to produce FPP-filled and FPP-based films for active and intelligent packaging. These two film types are typically produced by solvent casting, knife coating, or extrusion, with solvent casting being the most frequently adopted method. Despite this, knife coating and extrusion are more suitable for producing films at an industrial scale. FPP-filled films are characterized by uniform and compact inner structures, strong molecular interactions, low hydrophobicity, and high mechanical and barrier properties. In contrast, FPP-based films are generally characterized by heterogeneous and cracked inner structures, weak molecular interactions, high hydrophobicity, and low mechanical and barrier properties. Due to the presence of bioactive substances (e.g., polyphenols, essential oils, and pigments), both types of films are suitable for active and intelligent packaging. However, their commercialization is mainly limited by raw material variability, production unscalability, inherent performance flaws, and regulatory hurdles. Thus, future research is required to overcome these limitations.

## Figures and Tables

**Table 1:** The formulations, preparation methods, physical and functional properties, and applications of FPP-filled films.

Type of FPP	Particle Size of FPP	FPP Content	Polymers	Plasticizers (Content)	Reinforcing Agents	Preparation Methods of the Films	Impact of FPP on the Physical Properties of the Films	Functional Properties of the Films	Factors Affecting the Properties of the Films	Applications of the Films	References
Kinnow peel powder		5%, 10%, 15%, 20%, 25%	Soybean meal protein isolate	Glycerol (50%)		Solvent casting	Thickness ↑, MC ↓, WS ↓, SR ↓, WVP ↓, LT ↓, TS ↑, EAB ↑, YM ↑, thermal stability ↑	Antioxidant activity; Antimicrobial activity	The content of FPP		[13]
Pomegranate peel powder	75 µm	10%	Corn starch	Glycerol (20%)		Solvent casting		Antioxidant activity; Antimicrobial activity	The condition of homogenization	Active packaging for fresh-cut strawberry through wrapping	[16]
Sweet lime peel powder	150 µm		Gum Arabic/starch composite	Glycerol		Solvent casting					[17]
Grapefruit peel powder		2.5%, 5%	PVA	Glycerol (30%)	V_2_O_5_ nanoparticles	Solvent casting	Thickness ↑, MC ↓, WS ↓, WVP ↓, thermal stability ↑				[18]
Quince peel powder	250 µm		Potato starch	Glycerol		Solvent casting				Active packaging for cured cheese through wrapping	[19]
Dragon fruit peel powder	150 µm		Konjac glucomannan/kappa carrageenan composite	Glycerol		Solvent casting					[21]
Feijoa peel powder	75 µm	8%, 20%, 40%, 60%, 80%	Pinhão starch/citric pectin composite	Glycerol (40%)		Solvent casting	Thickness ↑, WS ↓, WCA ↔, LT ↓, TS ↔, EAB ↔, YM ↑, thermal stability ↑	Antioxidant activity; Antimicrobial activity	The content of FPP		[22]
Avocado peel powder	300 µm		Pectin	Polyglycerine (30%)	CaCl_2_	Solvent casting		Antioxidant activity			[54]
Banana peel powder	333 µm	5%, 10%, 15%, 20%, 25%	PVA			Solvent casting	WS ↑, SR ↑, WVP ↑, LT ↓, TS ↑, EAB ↓, YM ↑, thermal stability ↓, biodegradability ↑		The content of FPP		[55]
Banana peel powder	25 µm	5%, 10%, 15%, 20%, 25%	Cellulose			Knife coating	TS ↑, EAB ↓, YM ↑, thermal stability ↑		The content of FPP		[56]
Blueberry and jaboticaba peel powder	150 µm	12.5%	Corn starch	Glycerol (30%)		Solvent casting		pH sensitivity	The type of FPP		[57]
Dragon fruit peel powder	106 µm	9.60%	Sodium alginate	Glycerol (30%)	MgO nanoparticles	Solvent casting	Thickness ↑, MC ↔, WS ↓, WVP ↓, WCA ↑, LT ↓, TS ↑, EAB ↔, thermal stability ↑, biodegradability ↓	Antioxidant activity	The presence of MgO nanoparticles		[58]
Dragon fruit peel powder	150 µm		Gelatin	Glycerol		Solvent casting					[59]
Dragon fruit peel powder	150 µm		Konjac glucomannan	Glycerol		Solvent casting		pH sensitivity		Intelligent packaging for monitoring chicken meat freshness	[60]
Dragon fruit peel powder			Thermoplastic starch/agar composite	Glycerol		Compression molding					[32]
Grape peel powder	75 µm	2%, 4%, 6%, 8%	k-Carrageenan/hydroxypropyl methylcellulose composite	Sorbitol (40%)		Solvent casting	Thickness ↑, LT ↓, TS ↓, EAB ↓,	pH sensitivity	The content of FPP	Intelligent packaging for monitoring pork freshness	[33]
Jackfruit peel powder	250–500 µm	10%, 20%, 30%, 40%	PLA		Thymol	Extrusion and compression molding	TS ↓, EAB ↓, YM ↑, thermal stability ↓, biodegradability ↑		The content of FPP		[61]
Kinnow peel powder		20%	Soybean meal protein isolate	Glycerol (50%)	Montmorillonite	Solvent casting		Antioxidant activity; Antimicrobial activity	The content of montmorillonite		[62]
Kurugua peel powder	180 µm	6.25%, 12.5%	Cassava starch	Glycerol (30%)		Solvent casting	Thickness ↑, MC ↔, WS ↑, WCA ↑, LT ↓, thermal stability ↓	pH sensitivity	The content of FPP	Intelligent packaging for monitoring chicken meat freshness	[63]
Lychee peel powder	180 µm	2.5%, 5%, 7.5%, 10%	Chitosan	Glycerol (30%)		Solvent casting	Thickness ↑, MC ↓, WS ↓, SR ↓, WVP ↓, LT ↓, TS ↑, EAB ↓, YM ↑	Antioxidant activity; Antimicrobial activity	The content of FPP	Active packaging for fresh-cut apple and apple juice through wrapping	[64]
Mango peel powder	180 µm	40%, 80%	Corn starch	Sorbitol (40%)	Phosphoric acid	Solvent casting	TS ↑, EAB ↑	Antioxidant activity	The content of FPP	Active packaging for fresh-cut apple through edible coating	[65]
Mangosteen peel powder	180 µm	2.5%, 5%, 10%	Chitosan	Glycerol (30%)		Solvent casting	Thickness ↑, MC ↓, WS ↓, WVP ↑, LT ↓, TS ↑, EAB ↓, YM ↓, thermal stability ↑	Antioxidant activity; Antimicrobial activity	The content of FPP	Active packaging for soybean oil through wrapping	[66]
Orange peel powder	1 mm	20%, 30%, 40%, 50%	Corn starch	Glycerol (50%)		Solvent casting	Thickness ↔, MC ↓, WS ↓, SR ↑, TS ↑, EAB ↓, biodegradability ↑		The content of FPP		[67]
Orange, mandarin and lemon peel powder	40–50 µm	10%	PLA			Solvent casting	SR ↓, OP ↓, TS ↑, EAB ↓, YM ↑	Antioxidant activity	The type of FPP		[68]
Orange peel powder	90 µm	83%	LLDPE			Extrusion and compression molding		Antimicrobial activity			[69]
Orange peel powder	30–40 µm	5%, 10%, 15%, 20%	PVA			Solvent casting	thermal stability ↓		The content of FPP		[70]
Orange peel powder	100 µm	10%, 20%, 40%, 60%	PLA			Solvent casting	SR ↑, WCA ↑, TS ↓, EAB ↑, YM ↓, biodegradability ↑		The content of FPP		[71]
Orange peel powder	180 µm	3%, 6%, 9%, 12%, 15%	Gelatin	Glycerol (30%)		Solvent casting	Thickness ↑, MC ↑, WVP ↑, TS ↑, EAB ↓	Antioxidant activity; Antimicrobial activity	The content of FPP		[72]
Orange peel powder	100 µm	0.25%, 0.5%, 1%, 1.25%	Chitosan/PVA composite	Glycerol (20%)		Solvent casting	Thickness ↑, WS ↑, WCA ↓, WVP ↑, OP ↓, TS ↓, EAB ↑, YM ↔, thermal stability ↑	Antioxidant activity	The content of FPP		[34]
Orange peel powder		0.1%, 0.2%, 0.3%, 0.4%, 0.5%, 0.6%	Chitosan		ZnO nananoparticles	Solvent casting	Thickness ↑, SR ↓, TS ↑, EAB ↓, biodegradability ↓		The content of FPP and ZnO nananoparticles		[73]
Papaya peel powder	500 µm	2.5%, 5%, 7.5%	Gelatin			Solvent casting	MC ↔, WS ↓, WVP ↑, LT ↓, TS ↓, EAB ↓, YM ↓	Antioxidant activity	The micro-encapsulation of FPP	Active packaging for lard through wrapping	[74]
Papaya and citrus peel powder	25 µm	50%	Corn starch	Glycerol (75%)		Solvent casting			The ratio of two FPP	Active packaging for muffins	[75]
Pomegranate peel powder	35 µm	4%, 8%, 12%, 16%, 20%	PVA			Solvent casting	MC ↑, WVP ↓, OP ↓, thermal stability ↑		The content of FPP		[76]
Pomegranate peel powder	80 µm	2%, 4%, 6%, 8%, 10%, 12%, 14%	Hydroxypropyl high-amylose starch	Polyethylene glycol (20%)		Solvent casting	Thickness ↑, TS ↑, EAB ↓, YM ↑, LT ↓	Antimicrobial activity	The content of FPP		[77]
Pomegranate peel powder		2%, 4%, 6%, 8%	Cassava starch	Glycerol (30%)		Solvent casting	TS ↓, EAB ↓, YM ↓	pH sensitivity; Antioxidant activity	The content of FPP	Intelligent packaging for monitoring lamb meat freshness	[78]
Pomegranate, papaya and jackfruit peel powder		1%, 3%, 5%, 7%, 9%	Gelatin	Glycerol (30%)		Solvent casting	Thickness ↑, MC ↑, WS ↓, WCA ↑	Antioxidant activity; Antimicrobial activity	The type and content of FPP		[79]
Pomegranate peel powder		1%, 2%, 3%, 4%, 5%	Gelatin	Glycerol (30%)		Solvent casting	Thickness ↑, MC ↔, WS ↓, WVP ↑, TS ↑, EAB ↓	Antioxidant activity; Antimicrobial activity	The content of FPP		[80]
Pomegranate peel powder	80 µm		Polycaprolactone/starch composite		Stearic acid	Extrusion and compression molding		Antimicrobial activity			[35]
Pomegranate peel powder	13 µm		PLA/starch composite		Stearic acid and citric acid	Extrusion		Antimicrobial activity			[36]
Pomegranate peel powder		0.67%, 6.7%, 33%, 46.7%, 66.7%	Collagen/sodium alginate composite	Glycerol (0.1%, 1%, 5%, 7%, 10%)	Citric acid	Solvent casting		Antimicrobial activity	The content of FPP, glycerol and citric acid		[37]
Pomegranate peel powder		2.5%, 12.5%, 25%	Mung bean protein	Glycerol (50%)		Solvent casting	Thickness ↑, MC ↓, WS ↓, WCA ↑, WVP ↑, TS ↑, EAB ↑	Antioxidant activity; Antimicrobial activity	The content of FPP		[81]
Pomegranate peel powder	50 μm		PVA		Ag nanoparticles	Solvent casting		Antimicrobial activity	The content of Ag nanoparticles		[82]
Pomegranate peel powder	50 μm		PVA			Solvent casting	WVP ↓, TS ↑, EAB ↓, YM ↑, thermal stability ↑, biodegradability ↓	Antimicrobial activity	The content of FPP		[83]
Pomegranate peel powder	150 µm	3%, 6%, 9%	Chitosan	Glycerol (30%)		Solvent casting	Thickness ↑, MC ↓, WS ↑, WVP ↑, LT ↓, TS ↓, EAB ↓, YM ↓	Antioxidant activity; Antimicrobial activity	The content of FPP		[84]
Prickly pear peel powder		40%, 80%	Carboxymethyl cellulose	Glycerol (40%)		Solvent casting		Antioxidant activity			[85]
Sweet lime peel powder	106 µm	15%	PVA	Glycerol (20%)		Solvent casting	Thickness ↑, SR ↓, TS ↑, WVP ↓, thermal stability ↑, biodegradability ↑	Antimicrobial activity		Active packaging for bean sprout through covering	[86]
Orange peel powder	115 µm	25%, 43%, 67%, 100%	Corn starch	Glycerol (50%)		Solvent casting	Biodegradability ↑		The content of FPP		[87]
Orange peel powder		20%, 50%	Tara gum	Glycerol (30%, 50%)		Solvent casting	WS ↓, WVP ↑, TS ↑, EAB ↓		The contents of FPP and glycerol		[88]
Lemon and colocynth peel powder		2%	Wheat starch/PVA composite	Glycerol (30%)	Glutaraldehyde	Solvent casting		Antimicrobial activity	The type of FPP		[89]
Lemon peel powder	106 µm	1%, 2%, 3%, 4%	Hydroxyethyl cellulose/chitosan composite	Glycerol (25%)		Solvent casting	Thickness ↑, WS ↑, SR ↑, WVP ↑, TS ↓, EAB ↓	Antioxidant activity; Antimicrobial activity	The content of FPP	Active packaging for blueberries through wrapping	[90]
Mangosteen and durian peel powder	150 and 300 µm		PBAT			Extrusion and compression molding			The type and content of FPP		[91]
Dragon fruit peel powder	100 µm		PVA			Solvent casting			The content of FPP		[92]

EAB, elongation at break; FPP, fruit peel powder; LLDPE, linear low-density polyethylene; LT, light transmittance; MC, moisture content; OP, oxygen permeability; PBAT, polybutyrate adipate terephthalate; PLA, poly(lactic acid); PVA, polyvinyl alcohol; SR, swelling ratio; TS, tensile strength; WCA, water contact angle; WS, water solubility; WVP, water vapor permeability; YM, Young’s modulus; ↑, increased after FPP addition; ↓, decreased after FPP addition; ↔, unchanged after FPP addition. The contents of FPP and plasticizer are calculated based on the weight of polymer.

**Table 2:** The formulations, preparation methods, functional properties, and applications of FPP-based films.

Type of FPP	Particle Size of FPP	Polymers (Content)	Plasticizers (Content)	Reinforcing Agents	Preparation Methods of the Films	Functional Properties of the Films	Factors Affecting the Properties of the Films	Applications of the Films	References
Pomegranate and orange peel powder	150–125 µm, 106–75 µm, 75–53 µm, 53 µm		Glycerol (7% and 10%)	Citric acid	Solvent casting	Antioxidant activity; Antimicrobial activity	The particle size of FPP, the content of glycerol and the content of citric acid		[14]
Yellow peach peel powder	75 µm	Sodium alginate (24%)	Glycerol (32%)		Solvent casting	Antioxidant activity	The presence of sodium alginate and glycerol	Active packaging for soybean oil	[20]
Banana peel powder	500 µm	Carboxymethyl cellulose (20%)	Glycerol (15%)		Knife coating	Antioxidant activity; Antimicrobial activity	The pretreatment of FPP		[23]
Passion fruit peel powder	150 µm	Sodium alginate (10%)	Glycerol (30%)	Stearic acid	Solvent casting	Antioxidant activity; pH sensitivity	The presence of stearic acid	Intelligent packaging for monitoring shrimp freshness	[24]
Orange, lemon, pomelo and mandarin peel powder	150 µm	Sodium alginate (10%)	Glycerol (30%)		Solvent casting	Antioxidant activity; Antimicrobial activity	The variety of citrus fruits	Active packaging for corn oil through wrapping	[25]
Banana peel powder	355 µm	Corn starch (40%)	Glycerol (20%)	Banana leaf wax	Solvent casting		The presence of banana leaf wax		[45]
Dragon fruit peel powder	180 µm		Glycerol		Knife coating	Antioxidant activity; pH sensitivity	The content of FPP and glycerol	Intelligent packaging for monitoring pork freshness	[38]
Dragon fruit peel powder	150 µm	Cassava starch	Glycerol (30%)		Solvent casting	Antioxidant activity; Antimicrobial activity; pH sensitivity		Intelligent packaging for monitoring shrimp freshness	[39]
Lemon peel powder		Xanthan gum (0.25%, 0.5%, 0.75%)	Glycerol (20%)	TiO_2_–Ag nanoparticles	Solvent casting	Antioxidant activity; Antimicrobial activity			[46]
Mango and orange peel powder	180 µm	Sodium alginate (10%)	Glycerol; sorbitol	Aloe vera gel and essential oils	Solvent casting	Antimicrobial activity	The type of plasticizer and essential oils, and the addition of aloe vera gel	Active packaging for plum, grape and fresh-cut apple through edible coating	[43]
Orange, mango, banana, and sapodilla peel powder		Corn starch (100%)	Glycerol (100%) or Sorbitol (100%)		Solvent casting	Antimicrobial activity	The type of FPP and plasticizer		[44]
Orange peel powder	180 µm		Glycerol (30% based on FPP)	Wheat straw and rice husk powder	Solvent casting	Antioxidant activity; Antimicrobial activity	The content of wheat straw and rice husk powder		[47]
Orange peel powder	75 µm	Xanthan gum (25%, 50%, 75%, 100%)	Glycerol (50%, 100%, 200%, 300%)		Solvent casting		The content of Xanthan gum and glycerol		[48]
Orange peel powder	150 µm	Sodium alginate (30%)	Glycerol (30%)		Solvent casting	Antioxidant activity; Antimicrobial activity	The cultivar of orange	Active packaging for corn oil through wrapping	[49]
Pomegranate peel powder	500 µm	Silk fibroin (43%)	Glycerol (9%)		Solvent casting	Antioxidant activity; Antimicrobial activity			[41]
Pomegranate and orange peel powder	500 µm		Glycerol (20%, 25%, 30%)	CaCl_2_	Solvent casting		The proportion of pomegranate and orange peel powder	Active packaging for bread through wrapping	[42]
Pomelo peel powder	75–125 µm		Glycerol (2%, 4%, 6%)	Citric acid and AEAPTMS	Solvent casting		The kind of hydrophobic agent		[50]
Pomelo peel powder	150 µm	Sodium alginate (5%)	Glycerol (10%)	Tea polyphenol	Solvent casting	Antioxidant activity; Antimicrobial activity	The presence of tea polyphenol	Active packaging for soybean oil through wrapping	[51]
Sweet lime peel powder	500 µm	PVA (100%)	Glycerol (500%)	Sugarcane bagasse fiber	Solvent casting		The content of sugarcane bagasse fiber		[93]
Sweet lime peel powder	500 µm	PVA and starch	Glycerol (250%)		Solvent casting				[94]
Orange peel powder	100 µm	PLA (14%, 20%, 33%)			Solvent casting		The content of PLA		[52]
Orange peel powder		Gum Arabic (1%, 2%, 3%, 4%, 5%)	Glycerol (40%)	Cr_2_O_3_ nanoparticles	Solvent casting	Antimicrobial activity	The contents of gum Arabic and Cr_2_O_3_ nanoparticles		[53]
Dragon fruit peel powder		Cassava starch (1.5%, 3%, 4.5%, 6%, 7.5%, 9%)	Sorbitol (5.7%)	Eggshell powder	Solvent casting	Antioxidant activity	The content of cassava starch	Intelligent packaging for monitoring steamed chicken freshness	[40]
Prickly pear peel powder		Gelatin (100%)	Glycerol (50%)		Solvent casting	Antioxidant activity			[95]

AEAPTMS, *N*-(2-amino-ethyl)-3-aminopropyltrimethoxysilane; FPP, fruit peel powder; PLA, poly(lactic acid); PVA, polyvinyl alcohol. The contents of polymers and plasticizers are calculated based on the weight of the FPP.

**Table 3:** Comparison of the performance of FPP-filled and FPP-based films.

Film Performance	FPP-Filled Films	FPP-Based Films
Microstructure	Uniform and compact	Heterogeneous and cracked
Molecular interactions	Strong	Weak
Hydrophobicity	Low or high (depend on polymer matrix)	Medium
Mechanical properties	High	Low
Light barrier property	High	High
Water vapor and oxygen barrier properties	High	Low
Antioxidant and antimicrobial activities	Medium	High
pH sensitivity	Medium	High
Biodegradation	Slow or quick (depend on polymer matrix)	Quick
Functional stability	High	Low

## Data Availability

No new data were created or analyzed in this study. Data sharing is not applicable to this article.

## Supplementary Materials

The following supporting information can be downloaded at https://www.mdpi.com/article/10.3390/foods15010162/s1, Table S1: The proximate composition and functional components in different types of FPP.
