# Supplementary material for: Recent Advances in the Development of Active and Intelligent Packaging Films Using Fruit Peel Powders
Source: Foods. 2026 Jan 3;15(1):162. doi: 10.3390/foods15010162 (PMC12785629; doi:10.3390/foods15010162)
Supplement: Supplementary file 1 [file foods-15-00162-s001.zip › foods-4059395-supplementary.pdf]

Supplementary data:

Table S1. The proximate composition and functional components in different types of FPP.

Type of FPP	Proximate composition of FPP	Functional components in FPP	References
Pomegranate peel powder	Total dietary fiber (22%), pectin (2%), cellulose (11%), hemicellulose (6%), lignin (4%)	Polyphenols (e.g., punicalin, pedunculagin, punicalagin, ellagic acid, catechins, anthocyanins)	[16]
Sweet lime peel powder	Ash (3%), crude fiber (3%), fat (2%), protein (2%)	Polyphenols (e.g., hesperidin, luteolin, eriocitrin), essential oils (e.g., limonene, bergamol, β-pinene, linalool, α-pinene), carotenoids (e.g., β-carotene and lutein)	[17]
Grapefruit peel powder	Ash (5%), carbohydrate (70%), fiber (9%), fat (8%), protein (10%)	Polyphenols (e.g., naringin, hesperetin, narirutin, naringenin, isonaringin), essential oils (e.g., limonene, β-phellandrene, β-myrcene), carotenoids (e.g., phytoene and lycopene)	[18,28]
Quince peel powder	Starch (12%), pectin (2%), protein (2%), lipids (1.5%), fiber (5%)	Polyphenols (e.g., caffeoylquinic acid, kaempferol, quercetin)	[19]
Yellow peach peel powder	Pectin (27%), protein (9%), fat (5%), total dietary fiber (52%)	Polyphenols (e.g., chlorogenic acid, kaempferol, quercetin)	[20]
Dragon fruit peel powder	Ash (16%), fat (1%), pectin (23%), protein (10%)	Polyphenols (e.g., gallic acid, myricetin, quercetin), betacyanins	[21,29]
Feijoa peel powder	Ash (3%), carbohydrate (80%), fiber (9%), fat (3%), protein (5%)	Polyphenols (e.g., ferulic, syringic, coumaric and cinnamic acids), essential oils (e.g., sesquiterpenes)	[22]
Banana peel powder	Ash (2%), protein (5%), cellulose (17%), hemicellulose (6%), lignin (10%)	Polyphenols (e.g., gallic acid, catechin, tannins), carotenoids (e.g., lutein, γ-carotene, α-carotene, violaxanthin)	[23,30]
Passion fruit peel powder	Ash (13%), crude fiber (11%), pectin (5%), fat (4%), protein (14%)	Polyphenols (e.g., gallic acid, chlorogenic acid, catechins, anthocyanins), carotenoids (e.g., β-carotene and lutein)	[24,26]
Lemon peel powder	Ash (5%), crude fiber (3%), pectin (21%), fat (3%), protein (6%)	Polyphenols (e.g., naringin, hesperidin, eriocitrin), essential oils (e.g., limonene, β-pinene, γ-terpinene), carotenoids (e.g., violaxanthin, luteoxanthin, lutein, β-criptoxanthin)	[25]

Mandarin peel powder	Ash (3%), crude fiber (6%), pectin (24%), fat (7%), protein (7%)	Polyphenols (e.g., naringenin, hesperetin, neoponcirin, didymin, isorhoifolin, eriocitrin), essential oils (e.g., limonene, linalool, γ-terpinene, β-myrcene), carotenoids (e.g., β-cryptoxanthin, phytoene, β-criptoxantin, violaxantin)	[25,27]
Orange peel powder	Ash (4%), crude fiber (3%), pectin (24%), fat (8%), protein (8%)	Polyphenols (e.g., hesperetin, naringin), essential oils (e.g., limonene, β-myrcene, α-pinene), carotenoids (e.g., lutein, zeaxanthin, β-cryptoxanthin, β-carotene)	[25]
Pomelo peel powder	Ash (3%), crude fiber (6%), pectin (16%), fat (6%), protein (8%)	Polyphenols (e.g., naringenin, hesperetin, eriocitrin, narirutin, neohesperidin), essential oils (e.g., limonene, myrcene, γ-terpinene), carotenoids (e.g., phytoene, phytofluene, β-cryptoxanthin, zeaxanthin, α-carotene, β-carotene, lutein)	[25,31]

The proximate composition of FPP is expressed on a dry-weight basis of fruit peels.
